# Mortality prediction using the Shock Index and Modified Shock Index in the Emergency Department

**DOI:** 10.1590/0034-7167-2025-0257

**Published:** 2026-08-21

**Authors:** Karoline Maciel Lopes, Marcos Barragan da Silva, Juciane Aparecida Furlan Inchauspe, Cristiano Rossa da Rocha, Débora Ribas Leal, Tais Hochegger, Isabelle Muradás Paim, Michelle Dornelles Santarem

**Affiliations:** I Universidade Federal do Rio Grande do Sul. Porto Alegre, Rio Grande do Sul, Brazil; II Serviço de Emergência do Hospital de Clínicas de Porto Alegre. Porto Alegre, Rio Grande do Sul, Brazil

**Keywords:** Shock, Mortality, Emergency, Shock Index, Modified Shock Index., Choque, Mortalidad, Emergencia, Índice de Choque Modificado, Índice de Choque.

## Abstract

**Objectives:**

to compare the Shock Index and the Modified Shock Index in predicting in-hospital mortality among patients diagnosed with shock and admitted to the Emergency Department.

**Methods:**

a retrospective cohort (2022), including patients ≥18 years. The predictive ability was estimated using areas under the curve, sensitivity, and specificity, considering shock etiologies.

**Results:**

365 patients were included, (25.5 cases per 1,000 visits), mortality (75.3%), with a worse initial hemodynamic profile. Septic shock (69%) and hypovolemic shock (13.2%) predominated. The Shock Index demonstrated better areas under de curve in mixed (0.706) and septic (0.700) shock. The Modified Shock Index showed superiority in mixed (0.735), septic (0.700), and cardiogenic (0.700) shock, standing out in septic shock (sensitivity 96.0%; specificity 86.3%).

**Conclusions:**

the Modified Shock Index demonstrated greater predictive ability for mortality, suggesting its usefulness in nursing clinical practice.

## RESUMEN

**Objetivos:**

comparar el Índice de Shock y el Índice de Shock Modificado en la predicción de la mortalidad intrahospitalaria en pacientes con diagnóstico de shock admitidos en el Departamento de Emergencias.

**Métodos:**

cohorte retrospectiva (2022), que incluyó a pacientes ≥18 años. La capacidad predictiva se estimó mediante áreas bajo la curva, sensibilidad y especificidad, considerando las etiologías de shock.

**Resultados:**

se incluyeron 365 pacientes (25,5 casos por cada 1.000 atenciones, con una mortalidad (75,3%), con peor perfil hemodinámico inicial. Predominaron el shock séptico (69%) y el hipovolémico (13,2%). El Índice de Shock mostró mejores areas bajo la curva en los shocks mixto (0,706) y séptico (0,700). El Índice de Shock Modificado presentó un desempeño superior en los shocks mixto (0,735), séptico (0,700) y cardiogénico (0,700), destacándose en el séptico (sensibilidad 96,0%; especificidad 86,3%).

**Conclusiones:**

el Índice de Shock Modificado mostró una mayor capacidad predictiva para la mortalidad, lo que sugiere su utilidad en la práctica asistencial de enfermería.

## INTRODUCTION

Shock is a clinical syndrome characterized by the inability of the cardiovascular system to maintain an adequate supply of oxygen and nutrients to the tissues to meet metabolic demands. It can be classified as hypovolemic, distributive, cardiogenic, obstructive, or mixed shock (a combination of more than one type). The resulting hypoxia may lead to cellular death, target-organ injury, multiple organ failure, and ultimately the patient’s death if not properly treated^(1)^. Despite advances driven by evidence-based practice in the care of patients in shock, mortality remains high, reaching rates of up to 50%. Early recognition and immediate management of shock are the only variables proven to effectively reduce mortality, regardless of the type involved^(2)^.

In this context, tools that support the early identification of hemodynamic instability become essential for clinical decision-making. Introduced in 1967, the Shock Index (SI) is a score obtained by dividing heart rate (HR) by systolic blood pressure (SBP), providing a comprehensive assessment of hemodynamic status. This index enables early detection of hypovolemic shock and its severity, assists in identifying the need for massive transfusion, and predicts a higher risk of mortality^(3)^. Studies have demonstrated the association of SI with sepsis, cardiac complications, septic and cardiogenic shock, as well as with mortality risk. However, there is wide variability in cutoff points, which require adjustments to fit specific clinical contexts^(4,5)^.

The Modified Shock Index (MSI), an adaptation of the SI, is defined as the ratio between HR and mean arterial pressure (MAP). Evidence indicates that MSI is a more effective predictor of mortality when compared with SI alone. This modification is based on the fact that diastolic blood pressure decreases earlier in critically ill patients than systolic blood pressure, making MAP a more accurate marker for assessing clinical severity and indicating the need for emergency interventions^(6,7)^.

Despite the growing interest in using scoring tools for early identification and mortality prediction in patients with shock, there is still a scarcity of studies directly comparing predictive performance across different shock types, particularly in the emergency setting.

Considering the complexity of care in emergency units - marked by high demand, unpredictability, and often limited resources - work overload may lead to delays in managing patients at risk of shock and in administering interventions such as fluid resuscitation, antibiotic therapy, and hemodynamic support, ultimately increasing mortality^(8)^. In this scenario, even with therapeutic advances, there remains a clear need for simple, rapid, and reliable tools to support clinical decision-making in emergency services. Thus, the following research question arises: What is the predictive ability of the Modified Shock Index (MSI), compared with the Shock Index (SI), for in-hospital mortality among adult patients with different types of shock admitted to an emergency unit?

## OBJECTIVES

To compare the Shock Index (SI) and the Modified Shock Index (MSI) in predicting in-hospital mortality among patients diagnosed with shock and admitted to the Emergency Service.

## METHODS

### Ethical aspects

This study is part of a larger project entitled “Clinical outcomes and nursing care management of critically ill adult patients: A multicenter study,” approved by the Research Ethics Committee of the *Hospital de Clínicas de Porto Alegre* under opinion no. 4.131.571. The research complied with Resolution 466/2012 of the *Conselho Nacional de Saúde* (Brazilian National Health Council) and with the guidelines of the General Data Protection Law (LGPD), ensuring anonymity and confidentiality of the information

### Study design, period, and setting

This was a retrospective cohort study conducted between January and December 2022 in the Emergency Service of a university hospital located in southern Brazil. The institution is accredited by the Joint Commission International, attesting to its compliance with international standards of quality and patient safety. The Emergency Service has 56 beds distributed across a medical decision unit, short-stay and intermediate-care areas, a stabilization bay, and a sector dedicated to the care of critically ill patients. The triage and risk classification process is conducted according to the Manchester Triage System (MTS)^(9)^. In this protocol, the use of hemodynamic indices such as the Shock Index (SI) and the Modified Shock Index (MSI) is not part of the routine initial assessment. The study followed the recommendations of the Strengthening the Reporting of Observational Studies in Epidemiology (STROBE) guidelines.

### Sample, inclusion, and exclusion criteria

All patients admitted during the study period, aged ≥18 years, of both sexes, and who presented SBP ≤ 90 mmHg or MAP ≤ 65 mmHg associated with a clinical diagnosis of shock recorded in the medical chart were included. Data were entered into Microsoft Excel^®^ spreadsheets designed for the study. No exclusion criteria were established.

### Study protocol

Data were collected retrospectively by previously trained researchers through review of electronic medical records using a standardized instrument. Sociodemographic variables (age, sex, education level, marital status, employment status, and place of origin) and clinical variables (initial vital signs, type of shock, triage flowchart, risk classification, and length of stay) were included. Information was extracted from the hospital’s clinical database and organized into a specific spreadsheet for analysis, with data collection carried out between May and September 2025. Until that period, the service did not use mortality prediction scores in its routine; therefore, the scores analyzed were calculated exclusively for this study. When necessary, additional data were obtained through further review of the electronic medical record.

All records from admission to the emergency department until hospital discharge, transfer, or death were considered. The primary outcome was in hospital mortality, and the secondary outcomes were prolonged hospital stay (≥7 days) and the need for ICU admission. The 7 day cutoff was adopted due to its wide use in the literature to define long length of stay and because it corresponds to the median length of stay reported in national and international studies in the emergency setting.

The scores analyzed were the SI, calculated as the ratio between HR and SBP, and the MSI, obtained as the ratio between HR and MAP. Both were applied to eligible patients at the time of admission. The cutoff points used were based on the literature, and additional values were tested to identify the parameters with the best performance in predicting in hospital mortality.

### Analysis of results and statistical methods

Statistical analysis was performed using SPSS® software, version 23.0. Categorical variables were presented as absolute and relative frequencies and proportions and compared using the Chi-square or Fisher’s Exact tests. Continuous variables were described as mean and standard deviation, or median and interquartile range [IQR: Q1-Q3], according to their distribution assessed by the Shapiro-Wilk test and compared using Student’s t-test or the Mann-Whitney U test.

The predictive ability of SI and MSI was compared through ROC curve analysis, with estimation of the areas under the curve (AUC), sensitivity, and specificity for each type of shock. Score performance was interpreted based on AUC values, considering predictive those greater than 0.70. The significance level adopted for all analyses was p < 0.05.

## RESULTS

During the study period, 14,301 patients were admitted to the Emergency Service; 2,689 presented hypotension and, after applying the eligibility criteria, 365 (2.6%) were diagnosed with shock, resulting in an incidence of 25.5 cases per 1,000 visits. The sample consisted predominantly of men (54.8%), White individuals (85.5%), and had a median age of 66 years (IQR 56-74). Low educational level (60.8%), absence of a partner (59.5%), employment (51.5%), and residence in Porto Alegre (56.4%) were the most frequent characteristics. The most common triage flowchart was “Adult Malaise” (29.6%), and the predominant risk classification was “Very Urgent” (59.7%). Prolonged hospitalization occurred in 211 patients (57.8%), and 142 (38.9%) required ICU admission. In-hospital mortality was high (275; 75.3%), with 133 deaths (48.3%) occurring within the first seven days.

Septic shock was the most prevalent type (252; 69%), followed by hypovolemic (48; 13.2%), cardiogenic (39; 10.7%), and mixed shock (24; 6.6%), with the cardiogenic-septic combination being the most common within mixed cases (62.5%). The highest mortality rates occurred in septic shock (73.1%) and mixed shock (70.8%). Patients who died showed greater need for ICU admission (47.3% vs. 13.3%), higher frequencies of prolonged hospitalization (52.4% vs. 74.4% among survivors), and more compromised hemodynamic parameters: higher heart rate (101.74 vs. 93.30 bpm), higher SI (1.31 vs. 1.19), and higher MSI (1.78 vs. 1.58). (Table 1)

**Table 1:** Clinical characteristics of patients admitted to the Emergency Service in 2022, Porto Alegre, Rio Grande do Sul, Brazil

Variables	Covariates	Total		Death (YES)		Death (NO)		*p* value
		N=365	100 (%)	N=275	75.3 (%)	N=90	24.7 (%)	
**Flow Chart**	Malaise	108	29.6	84	30.5	24	26.7	0.718^**^
	Adult dyspnea	80	21.9	63	22.9	17	18.9	
	Chest pain	34	9.3	25	9.1	9	10	
	Adult abdominal pain	33	9.0	26	9.5	7	7.8	
	Unclassified	38	10.4	26	9.5	12	13.3	
	Others	72	19.7	51	18.5	21	23.3	
**Severity**	Emergency (Red)	62	17	42	15.3	20	22.2	0.254^**^
	Very Urgent (Orange)	218	59.7	171	62.2	47	52.2	
	Urgent (Yellow)	47	12.9	36	13.1	11	12.2	
	Unclassified (White)	38	10.4	26	9.5	12	13.3	
**ICU Admission**	Yes	142	38.9	130	47.3	12	13.3	<0.001^**^
**Prolonged hospitalization (> 7 d)**	Yes	211	57.8	144	52.4	67	74.4	<0.001^**^
**Length of stay (days)**	Median (IQR)	9 (2.5;20)		8 (2;19)		12 (4;27.25)		<0.001^*^
**Types of Shock**	Septic	252	69	200	73.1	51	56.7	<0.001^**^
	Hypovolemic	48	13.2	27	9.8	21	23.3	
	Cardiogenic	39	10.7	30	10.9	9	10	
	Mixed shock	24	6.6	17	6.2	7	7.8	
	Others	2	0.5	0	0	2	2.2	
**Clinical Data**	Systolic Blood Pressure			79 (68;87)		81 (76;88)		0.192^*^
	Diastolic Blood Pressure			46 (39;52)		48 (42;54)		0.103^*^
	Mean Arterial Pressure			57(49.33;63.33)		60 (54.58;64)		0.121^*^
	Heart Rate			101.74 (27.96)		93.30 (30.41)		0.018^***^
	Shock Index			1.31 (1.04;1.68)		1.19 (0.89;1.41)		0.014^*^
	Modified Shock Index			1.78 (1.44;2.29)		1.58 (1.23;1.92)		0.023^*^

* Mann-Whitney U test;

** Pearson’s Chi-square test;

*** Student’s t-test.

**Figure 1:**
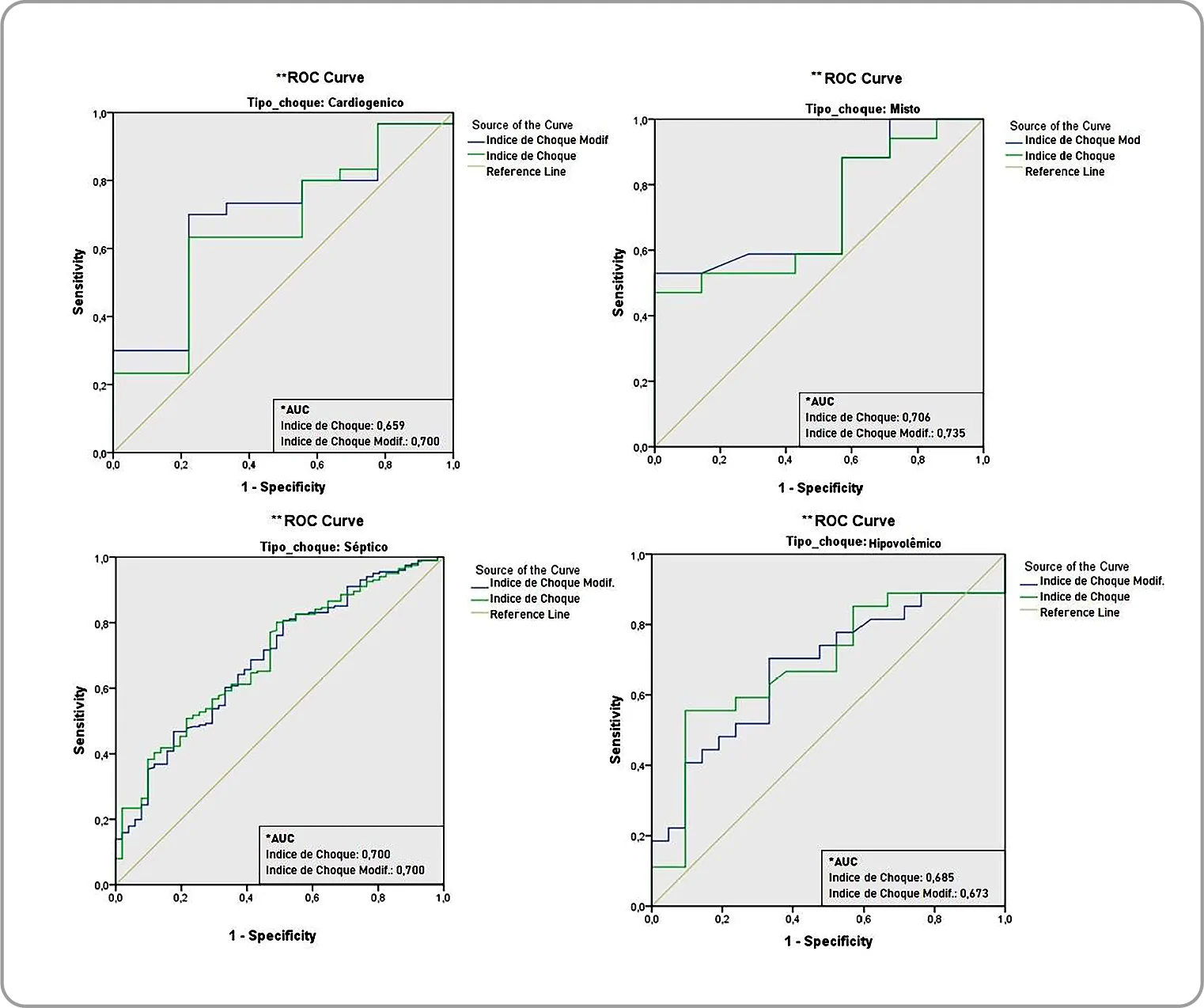
Comparison of the areas under the ROC curves (AUC) of the Shock Index and Modified Shock Index for predicting in-hospital mortality in patients diagnosed with shock, Porto Alegre, Rio Grande do Sul, Brazil

In the predictive performance analysis, the SI showed better performance in mixed shock (AUC 0.706) and septic shock (AUC 0.700). The MSI demonstrated superior performance in mixed shock (AUC 0.735), septic shock (AUC 0.700), and cardiogenic shock (AUC 0.700). In septic shock, the MSI showed a sensitivity of 96.0% and specificity of 86.3%, outperforming the SI (82.1% and 54.9%). In mixed shock, the MSI showed a sensitivity of 94.1% and specificity of 71.4%, also superior to the SI (76.5% and 57.1%). In cardiogenic shock, the MSI achieved a sensitivity of 90.0% and specificity of 77.8%, surpassing the SI (63.3% and 22.2%). In hypovolemic shock, performances were similar, with the SI showing sensitivity of 88.9% and specificity of 71.4%, compared with 81.5% and 66.7% for the MSI. Overall, the MSI demonstrated more consistent performance, particularly in septic shock, which was the most prevalent type. (Table 2 and Figure 1)

**Table 2:** Predictive ability of the Shock Index and Modified Shock Index for in-hospital mortality, according to shock type, in patients admitted to the Emergency Service, Porto Alegre, Rio Grande do Sul, Brazil, 2022

Type of Shock	Scores assessed	Cutoff point ^*^	Sensitivity (%)	Specificity (%)	AUC^†^	CI ^**^ 95%	*p* value
Cardiogenic	SI ^‡^	>1.0	63.3	22.2	0.659	0.46-0.86	0.152
	MSI ^‡‡^	>1.1	90.0	77.8	**0.700** ^††^	0.51-0.89	0.072
Septic	SI ^‡^	> 1.0	82.1	54.9	**0.700** ^††^	0.62-0.77	**<0.001** ^††^
	MSI ^‡‡^	>1.0	96.0	86.3	**0.700** ^††^	0.61-0.77	**<0.001** ^††^
Hypovolemic	SI ^‡^	> 1.4	88.9	71.4	0.685	0.53-0.84	**0.029** ^††^
	MSI ^‡‡^	> 1.9	81.5	66.7	0.673	0.52-0.83	**0.042** ^††^
Mixed	SI ^‡^	> 1.1	76.5	57.1	**0.706** ^††^	0.49-0.92	0.120
	MSI ^‡‡^	>1.2	94.1	71.4	**0.735** ^††^	0.52-0.94	0.075

* The Youden test was used to determine the optimal cutoff point; ‡SI - Shock Index; ‡‡MSI - Modified Shock Index; †AUC - area under the curve;

** CI - confidence interval; ††Statistically significant.

* AUC - Area Under the Curve;

** ROC - Receiver Operating Characteristic.

## DISCUSSION

The comparison between the Shock Index (SI) and the Modified Shock Index (MSI) showed that both are capable of predicting in hospital mortality among patients with shock admitted to the Emergency Service; however, the MSI demonstrated more consistent performance, particularly in the most prevalent shock types, thus addressing the central objective of this study.

The literature on the incidence of shock remains limited. A cohort study conducted in an Emergency Department in Thailand reported an incidence of 876 cases among 113,651 individuals (7.7 per 1,000 visits), with septic shock being the most prevalent, followed by hypovolemic and cardiogenic shock. In hospital mortality was assessed at two time points: 7.9% of deaths occurred within 7 days, and 20.4% within 90 days. At the latter time point, higher mortality rates were observed among patients with hypovolemic shock, followed by cardiogenic and septic shock^(10)^. The incidence and in hospital mortality rates observed in the present study were higher than those described in the international literature, which may be partially explained by the profile of the institution where the research was conducted. It is a university hospital that predominantly serves patients from the “SUS” (Brazilian Unified Health System), many of whom face barriers to accessing healthcare services, have limited outpatient follow up, and receive fewer preventive and continuous monitoring interventions. This scenario contributes to patients arriving at the emergency department in more advanced stages of hemodynamic deterioration, often with multiple decompensated comorbidities, severe infections, or significant dehydration, increasing the complexity of care and the risk of mortality.

Despite the differences in mortality outcomes, the distribution of shock etiologies observed in our sample is consistent with previous studies, reinforcing that septic shock remains the leading cause in emergency services, followed by hypovolemic and cardiogenic causes.

Additionally, older adults, men, and White individuals showed greater susceptibility to developing shock. Two cohort studies conducted in emergency departments - a retrospective cohort of 876 medical patients in Thailand and a 12-year cohort in Denmark including 1,553 patients - also found that this patient profile is more vulnerable to developing shock^(10,11)^.

The predominance of older adults in emergency services may be partially attributed to the ongoing demographic transition in Brazil, characterized by a substantial increase in the elderly population. This age group presents a higher burden of comorbidities, greater predisposition to cardiovascular events, and increased vulnerability to infectious processes - factors that significantly elevate the risk of developing shock, as well as in-hospital mortality rates, length of stay, and the direct and indirect costs associated with healthcare resource utilization^(12,13)^. In this context, initial management plays a central role, especially when clinical manifestations present as nonspecific complaints, requiring professionals to recognize early signs of hemodynamic instability and apply specific assessment and intervention protocols^(14,15)^. In services that adopt risk-classification systems, the role of nurses is particularly noteworthy in the early identification of risk factors, enabling the appropriate assignment of clinical discriminators, prioritization of care, and timely implementation of interventions that minimize the progression of shock and its complications.

The present study showed that the SI demonstrated better predictive ability for in-hospital mortality in mixed and septic shock, whereas the MSI exhibited superior performance in mixed, septic, and cardiogenic shock. In line with these findings, a cohort study conducted with 200 patients in an Italian ICU identified higher mortality among individuals with mixed shock (53.1%) compared with those with single-cause shock (27.8%), a difference attributed to greater pathophysiological complexity^(16)^. In the present study, this lethality was even higher, reaching 70% among patients with mixed shock. For cardiogenic shock, previous research has reported mortality rates ranging from 27% to 51%^(17,18)^, values lower than those observed in our sample, possibly due to a greater burden of comorbidities, increased frailty, and reduced physiological reserve - factors that compromise the compensatory mechanisms required to reverse refractory shock.

Despite the clinical relevance of these findings, the literature still lacks studies that specifically evaluate the performance of the SI and MSI in mixed shock. In patients with cardiovascular diseases, evidence shows an association between SI ≥ 0.8 and severe complications, such as cardiogenic shock^(18)^, whereas MSI ≥ 0.9 has proven effective in predicting mortality in acute myocardial infarction^(19)^. However, the cutoff points identified in the present study for optimal predictive performance in cardiogenic shock were higher (SI ≥ 1.0; MSI ≥ 1.1), which may reflect differences in the characteristics of the populations analyzed, the care profiles, and the severity of the cases treated.

Similarly, septic shock showed high mortality in our sample. National studies report lethality of 53.5%^(20)^, whereas in the present study the rate reached 80%. Previous research has reported effective performance of the SI and MSI with cutoff values of 0.76 and 1.35, respectively^(21)^, or SI ≥ 1.06 and MSI ≥ 1.64^(22)^. In contrast, the findings of this study indicated a lower cutoff point (≥ 1.0) as the most appropriate for both scores, possibly due to the more severe clinical profile of the patients treated.

In hypovolemic shock, the best cutoff points observed were ≥ 1.4 for the SI and ≥ 1.9 for the MSI. Although studies involving trauma populations suggest SI > 0.9 as an indicator of higher mortality^(5,23,24)^, analyses of larger cohorts have shown that SI > 1.4 is associated with unfavorable outcomes^(25)^. Regarding the MSI, values > 1.3 have been correlated with increased mortality in trauma^(26)^. Thus, the discrepancies observed across studies may be explained by population, etiological, and contextual differences.

Understanding the underlying physiology of these scores helps explain their performance. The MSI incorporates mean arterial pressure, more accurately reflecting tissue perfusion status compared with the SI. The early decline in diastolic blood pressure, captured by the MSI, allows earlier detection of hemodynamic instability, providing greater sensitivity for identifying the initial compensatory stages of shock^(27)^. This may explain the overall superior performance of the MSI observed in this study, with the exception of hypovolemic shock.

Finally, these findings highlight the potential of the MSI as a clinically relevant tool in nursing assessment. Its incorporation into initial evaluation or risk classification protocols may assist nurses in the early identification of patients at increased risk of clinical deterioration, optimizing care prioritization and decision making from the moment of triage. Moreover, this study stands out as one of the first to comparatively evaluate the performance of the SI and MSI in a large cohort of Brazilian patients treated in an emergency service, reinforcing the usefulness of simple, low cost tools in high demand, resource limited settings.

### Study limitations

This study presents limitations inherent to its retrospective design, particularly the dependence on the quality of electronic medical record documentation, which may have introduced information bias due to missing data in some cases. However, the sample size and the robust statistical analysis provide relevance and consistency to the results obtained. Caution is recommended when generalizing these findings, and multicenter prospective studies are needed to validate the identified cutoff points and confirm the applicability of these results in different clinical settings.

### Study contributions

The findings of this research contribute to strengthening the triad of education, care, and research by supporting the training of students and professionals and expanding understanding of the use of predictive scores within nursing practice. In addition, they offer practical implications for nursing in emergency services by demonstrating that these scores can be incorporated as rapid tools for triage and risk stratification, facilitating clinical reasoning, care prioritization, and efficient allocation of resources. These results may also support the development of flowcharts, protocols, and public policies aimed at reducing mortality from shock, as well as serve as a foundation for future validation studies in diverse clinical and population contexts.

## CONCLUSIONS

The Modified Shock Index (MSI) demonstrated superior ability to predict in-hospital mortality among patients admitted with shock, particularly in cases of septic, cardiogenic, and mixed shock. In contrast, the Shock Index (SI) showed better predictive performance only in hypovolemic shock. These results highlight the potential of these scores - especially the MSI - as simple and effective tools for triage and risk stratification in emergency services. The use of these scores may support clinical reasoning in nursing, facilitate rapid decision-making, and contribute to care prioritization in high-demand, resource-limited settings. The incorporation of these scores into clinical protocols is recommended, as well as the development of new multicenter prospective studies to validate the identified cutoff points and expand the applicability of shock indices across different clinical scenarios

## Data Availability

The research data are available only upon request.
