# Supplementary material for: Mortality prediction using the Shock Index and Modified Shock Index in the Emergency Department
Source: Rev Bras Enferm. 2026 Aug 21;79:e20250257. doi: 10.1590/0034-7167-2025-0257 (PMC13506213; doi:10.1590/0034-7167-2025-0257)
Supplement: Translation

# Translation: Predição de mortalidade do Índice de Choque e Índice de Choque Modificado na Emergência

**DOI:** 10.1590/0034-7167-2025-0257pt

**Authors:** Karoline Maciel Lopes, Marcos Barragan da Silva, Juciane Aparecida Furlan Inchauspe, Cristiano Rossa da Rocha, Débora Ribas Leal, Tais Hochegger, Isabelle Muradás Paim, Michelle Dornelles Santarem

**Affiliations:** I Universidade Federal do Rio Grande do Sul. Porto Alegre, Rio Grande do Sul, Brasil; II Serviço de Emergência do Hospital de Clínicas de Porto Alegre. Porto Alegre, Rio Grande do Sul, Brasil

**Keywords:** Choque, Mortalidade, Emergência, Índice de Choque, Índice de Choque Modificado.

## Abstract

**Objetivos:**

comparar o Índice de Choque e o Índice de Choque Modificado na predição de mortalidade intra-hospitalar em pacientes com diagnóstico de choque admitidos na Emergência.

**Métodos:**

coorte retrospectiva (2022), incluindo pacientes ≥18 anos. A capacidade preditiva foi estimada por áreas sob a curva, sensibilidade e especificidade, conforme as etiologias de choque.

**Resultados:**

incluídos 365 pacientes (25,5 casos por 1.000 atendimentos), mortalidade de 75,3%, com pior perfil hemodinâmico inicial. Predominaram choque séptico (69%) e hipovolêmico (13,2%). O Índice de Choque apresentou melhores áreas sob a curva nos choques misto (0,706) e séptico (0,700). O Índice de choque Modificado superou-o nos choques misto (0,735), séptico (0,700) e cardiogênico (0,700), destacando-se no séptico (sensibilidade 96,0%; especificidade 86,3%).

**Conclusões:**

o Índice de Choque Modificado apresentou maior capacidade preditiva para mortalidade, sugerindo sua utilidade na prática assistencial do enfermeiro.

## INTRODUÇÃO

O choque é uma síndrome clínica, caracterizada pela incapacidade do sistema cardiovascular em manter uma adequada oferta de oxigênio e nutrientes aos tecidos de forma a suprir as demandas metabólicas, podendo ser classificado em choque hipovolêmico, distributivo, cardiogênico, obstrutivo ou misto (combinação de mais de um tipo de choque). A hipóxia resultante pode culminar em morte celular, lesão de órgãos-alvo, falência múltipla de órgãos e, em última instância, levar ao óbito do paciente, caso não seja tratada adequadamente^(1)^. Apesar dos avanços proporcionados pela prática baseada em evidências no cuidado a pacientes em choque, a mortalidade permanece elevada, podendo atingir taxas de até 50%. O reconhecimento precoce e a abordagem imediata do estado de choque configuram-se como as únicas variáveis comprovadamente eficazes na redução da mortalidade, independentemente do tipo envolvido^(2)^.

Nesse contexto, ferramentas que favorecem a identificação precoce da instabilidade hemodinâmica tornam-se essenciais para a tomada de decisão clínica. Introduzido em 1967, o Índice de Choque (IC) é um escore obtido pela divisão entre a frequência cardíaca (FC) e a pressão arterial sistólica (PAS), fornecendo uma avaliação abrangente do estado hemodinâmico. Esse índice permite a detecção precoce do choque hipovolêmico e de sua gravidade, além de auxiliar na identificação da necessidade de transfusão maciça e na predição de maior risco de mortalidade^(3)^. Estudos demonstram a associação do IC com sepse, complicações cardíacas, choque séptico e cardiogênico, bem como com o risco de mortalidade. Entretanto, há ampla variabilidade nos pontos de corte, os quais necessitam de ajustes para se adequarem a contextos clínicos específicos^(4,5)^.

O Índice de Choque Modificado (ICM), uma adaptação do IC, é definido como a razão entre a FC e a pressão arterial média (PAM). Evidências apontam que o ICM é um preditor de mortalidade mais eficaz quando comparado ao IC isoladamente. Essa modificação baseia-se no fato de que a pressão arterial diastólica apresenta queda precoce em pacientes críticos em relação à pressão arterial sistólica, o que torna a PAM um marcador mais preciso para avaliar a gravidade clínica da doença e indicar a necessidade de intervenções emergenciais^(6,7)^.

Apesar do crescente interesse na utilização de escores para a identificação precoce e para a predição de mortalidade em pacientes com choque, ainda há escassez de estudos que comparem diretamente a capacidade preditiva entre diferentes tipos, especialmente no contexto da emergência.

Considerando a complexidade dos atendimentos nas unidades de emergência, marcadas por alta demanda, imprevisibilidade e recursos frequentemente limitados, a sobrecarga de trabalho pode resultar em atraso no atendimento nas situações de risco de choque e na administração de intervenções, como reposição volêmica, antibioticoterapia e suporte hemodinâmico, aumentando a mortalidade^(8)^. Diante desse cenário, mesmo com avanços terapêuticos, destaca-se a necessidade de ferramentas simples, rápidas e confiáveis para apoiar a tomada de decisão clínica nos serviços de emergência. Assim, surge a seguinte questão de pesquisa: Qual é a capacidade preditiva do Índice de Choque Modificado (ICM), em comparação ao Índice de Choque (IC), para a mortalidade intra-hospitalar de pacientes adultos com diferentes tipos de choque admitidos em unidade de emergência?

## OBJETIVOS

Comparar o Índice de Choque (IC) e o Índice de Choque Modificado (ICM) na predição de mortalidade intra-hospitalar em pacientes com diagnóstico de choque admitidos no Serviço de Emergência.

## MÉTODOS

### Aspecto ético

Este estudo integra o projeto maior intitulado “Desfechos clínicos e gestão da assistência de enfermagem do paciente adulto crítico: Estudo multicêntrico”, aprovado pelo Comitê de Ética em Pesquisa do Hospital de Clínicas de Porto Alegre, sob o parecer nº 4.131.571. A pesquisa atendeu a Resolução 466/2012 do Conselho Nacional de Saúde e às diretrizes da Lei Geral de Proteção de Dados (LGPD), assegurando anonimato e a confidencialidade das informações.

### Desenho, período, e local do estudo

Trata-se de um estudo de coorte retrospectivo, realizado entre janeiro e dezembro de 2022, em um Serviço de Emergência de um hospital universitário localizado no sul do Brasil. A instituição possui acreditação pela *Joint Commission International*, atestando o cumprimento de padrões internacionais de qualidade e segurança assistencial. O Serviço de Emergência dispõe de 56 leitos, distribuídos entre unidade de decisão médica, internação breve e intermediária, box de estabilização e setor destinado ao atendimento de pacientes críticos. O processo de acolhimento e classificação de risco é conduzido conforme o Sistema de Triagem de Manchester (STM)^(9)^. Nesse protocolo, o uso de índices hemodinâmicos como o Índice de Choque (IC) e o Índice de Choque Modificado (ICM) não integra a rotina de avaliação inicial. O estudo seguiu as recomendações do *Strengthening the Reporting of Observational Studies in Epidemiology* (STROBE).

### Amostra, critérios de inclusão e exclusão

Foram incluídos todos os pacientes admitidos no período do estudo, com idade ≥ 18 anos, de ambos os sexos, e que apresentaram PAS ≤ 90 mmHg ou PAM ≤ 65 mmHg, associadas ao diagnóstico clínico de choque registrado em prontuário cujos dados foram tabulados em planilhas do *Microsoft Excel* ^®^ projetadas para o estudo. Não foram estabelecidos critérios de exclusão.

### Protocolo do estudo

Os dados foram coletados retrospectivamente por pesquisadores previamente capacitados, mediante consulta aos prontuários eletrônicos e utilização de instrumento padronizado. Foram incluídas variáveis sociodemográficas (idade, sexo, escolaridade, estado civil, situação profissional e procedência) e clínicas (sinais vitais iniciais, tipo de choque, fluxograma de entrada, classificação de risco e tempo de internação). As informações foram extraídas da base de dados assistencial do hospital e organizadas em planilha específica para análise, com coleta realizada entre maio e setembro de 2025. Até esse período, o serviço não adotava escores de predição de mortalidade em sua rotina; portanto, os escores analisados foram calculados exclusivamente para este estudo. Quando necessário, dados complementares foram obtidos por meio da revisão do prontuário eletrônico.

Foram considerados todos os registros desde a admissão na emergência até alta hospitalar, transferência ou óbito. O desfecho principal foi a mortalidade intra-hospitalar, e os desfechos secundários foram tempo de internação prolongado (≥ 7 dias) e necessidade de internação em UTI. O ponto de corte de 7 dias foi adotado por sua ampla utilização na literatura para definição de *long-length of stay* e por corresponder à mediana de permanência observada em estudos nacionais e internacionais no cenário da emergência.

Os escores analisados foram O IC, calculado pela razão entre FC e PAS, e o ICM, obtido pela razão entre FC e PAM. Ambos foram aplicados aos pacientes elegíveis no momento da admissão. Os pontos de corte utilizados foram baseados na literatura, e valores adicionais foram testados para identificar os parâmetros com melhor desempenho na predição de mortalidade intra-hospitalar.

### Análise dos resultados e estatística

A análise estatística foi realizada no software SPSS^®^, versão 23.0. As variáveis categóricas foram apresentadas em frequências absolutas, relativas e proporções, sendo comparadas pelos testes Qui-quadrado ou Exato de Fisher. As variáveis contínuas foram descritas como média e desvio padrão, ou mediana e intervalo interquartil [IIQ: Q1-Q3], conforme a distribuição avaliada pelo teste de Shapiro-Wilk, e comparadas pelo teste t de *Student* ou Mann-Whitney U.

A comparação da capacidade preditiva do IC e do ICM foi realizada por meio da análise das curvas ROC, com estimativa das áreas sob a curva (AUC), sensibilidade e especificidade para cada tipo de choque. O desempenho dos escores foi interpretado com base nos valores de AUC, considerando-se preditivos aqueles superiores a 0,70. O nível de significância adotado para todas as análises foi de p < 0,05.

## RESULTADOS

Durante o período do estudo, 14.301 pacientes foram admitidos no Serviço de Emergência; 2.689 apresentaram hipotensão e, após aplicação dos critérios de elegibilidade, 365 (2,6%) foram diagnosticados com choque, resultando em uma incidência de 25,5 casos por 1.000 atendimentos. A amostra foi composta majoritariamente por homens (54,8%), brancos (85,5%) e com mediana de idade de 66 anos (IIQ 56-74). Predominaram baixa escolaridade (60,8%), ausência de companheiro (59,5%), atividade profissional (51,5%) e procedência de Porto Alegre (56,4%). O fluxograma mais frequente foi “Mal-estar em adulto” (29,6%), e a classificação de risco predominante foi “Muito Urgente” (59,7%). A internação prolongada ocorreu em 211 pacientes (57,8%) e 142 (38,9%) necessitaram de UTI. A mortalidade intra-hospitalar foi elevada (275; 75,3%), com 133 óbitos (48,3%) nos primeiros sete dias.

O choque séptico foi o mais prevalente (252; 69%), seguido dos choques hipovolêmico (48; 13,2%), cardiogênico (39; 10,7%) e misto (24; 6,6%), predominando no misto a combinação cardiogênico e séptico (62,5%). As maiores mortalidades ocorreram no choque séptico (73,1%) e no misto (70,8%). Pacientes que evoluíram para óbito apresentaram maior necessidade de UTI (47,3% vs. 13,3%), maiores frequências de internação prolongada (52,4% vs. 74,4%, entre sobreviventes) e parâmetros hemodinâmicos mais comprometidos: frequência cardíaca mais elevada (101,74 vs. 93,30 bpm), IC mais alto (1,31 vs. 1,19) e ICM mais elevado (1,78 vs. 1,58). (Tabela 1)

**Tabela 1:** Características referentes à variáveis clínicas dos pacientes admitidos no serviço de Emergência em 2022, Porto Alegre, Rio Grande do Sul, Brasil

Variáveis	Covariáveis	Total		Óbito (SIM)		Óbito (NÃO)		Valor de *p*
		N=365	100 (%)	N=275	75,3 (%)	N=90	24,7 (%)	
**Fluxograma**	Mal-estar	108	29,6	84	30,5	24	26,7	0,718^**^
	Dispneia em adulto	80	21,9	63	22,9	17	18,9	
	Dor torácica	34	9,3	25	9,1	9	10	
	Dor abdominal no adulto	33	9,0	26	9,5	7	7,8	
	Sem classificação	38	10,4	26	9,5	12	13,3	
	Outros	72	19,7	51	18,5	21	23,3	
**Gravidade**	Emergência (Vermelho)	62	17	42	15,3	20	22,2	0,254^**^
	Muito Urgente (Laranja)	218	59,7	171	62,2	47	52,2	
	Urgente (Amarelo)	47	12,9	36	13,1	11	12,2	
	Sem Classificação (Branco)	38	10,4	26	9,5	12	13,3	
**Internação em UTI**	Sim	142	38,9	130	47,3	12	13,3	<0,001^**^
**Internação prolongada (> 7 d)**	Sim	211	57,8	144	52,4	67	74,4	<0,001^**^
**Tempo de internação (em dias)**	Mediana (IIQ)	9 (2,5;20)		8 (2;19)		12 (4;27,25)		<0,001^*^
**Tipos de Choque**	Séptico	252	69	200	73,1	51	56,7	<0,001^**^
	Hipovolêmico	48	13,2	27	9,8	21	23,3	
	Cardiogênico	39	10,7	30	10,9	9	10	
	Choque misto	24	6,6	17	6,2	7	7,8	
	Outros	2	0,5	0	0	2	2,2	
**Dados Clínicos**	Pressão Arterial Sistólica			79 (68;87)		81 (76;88)		0,192^*^
	Pressão Arterial Diastólica			46 (39;52)		48 (42;54)		0,103^*^
	Pressão Arterial Média			57(49,33;63,33)		60 (54,58;64)		0,121^*^
	Frequência Cardíaca			101,74 (27,96)		93,30 (30,41)		0,018^***^
	Índice de Choque			1,31 (1,04;1,68)		1,19 (0,89;1,41)		0,014^*^
	Índice de Choque Modificado			1,78 (1,44;2,29)		1,58 (1,23;1,92)		0,023^*^

* Teste de Mann-Whitney-U;

** Qui-quadrado Pearson;

*** Teste T Student.

Na análise da capacidade preditiva, o IC apresentou melhor desempenho nos choques misto (AUC 0,706) e séptico (AUC 0,700). O ICM demonstrou desempenho superior no choque misto (AUC 0,735), séptico (AUC 0,700) e cardiogênico (AUC 0,700). No choque séptico, o ICM apresentou sensibilidade de 96,0% e especificidade de 86,3%, superando o IC (82,1% e 54,9%). No choque misto, o ICM mostrou sensibilidade de 94,1% e especificidade de 71,4%, também superior ao IC (76,5% e 57,1%). No cardiogênico, o ICM obteve sensibilidade de 90,0% e especificidade de 77,8%, superando o IC (63,3% e 22,2%). No hipovolêmico, os desempenhos foram próximos, com sensibilidade de 88,9% e especificidade de 71,4% para o IC e 81,5% e 66,7% para o ICM. De forma geral, o ICM apresentou desempenho mais consistente, sobretudo no choque séptico, que foi o tipo mais prevalente. (Tabela 2 e Figura 1)

**Tabela 2:** Capacidade preditiva do Índice de Choque e do Índice de Choque Modificado para mortalidade intra-hospitalar, segundo tipo de choque, em pacientes admitidos no Serviço de Emergência, Porto Alegre, Rio Grande do Sul, Brasil, 2022

Tipo de choque	Escores mensurados	Ponto de corte ^*^	Sensibilidade (%)	Especificidade (%)	AUC^†^	IC ^**^ 95%	Valor de *p*
Cardiogênico	IC ^‡^	>1,0	63,3	22,2	0,659	0,46-0,86	0,152
	ICM ^‡‡^	>1,1	90,0	77,8	**0,700** ^††^	0,51-0,89	0,072
Séptico	IC ^‡^	> 1,0	82,1	54,9	**0,700** ^††^	0,62-0,77	**<0,001** ^††^
	ICM ^‡‡^	>1,0	96,0	86,3	**0,700** ^††^	0,61-0,77	**<0,001** ^††^
Hipovolêmico	IC ^‡^	> 1,4	88,9	71,4	0,685	0,53-0,84	**0,029** ^††^
	ICM ^‡‡^	> 1,9	81,5	66,7	0,673	0,52-0,83	**0,042** ^††^
Misto	IC ^‡^	> 1,1	76,5	57,1	**0,706** ^††^	0,49-0,92	0,120
	ICM ^‡‡^	>1,2	94,1	71,4	**0,735** ^††^	0,52-0,94	0,075

* Para definição de melhor ponto de corte foi utilizado o teste de Youden; ‡IC - Índice de Choque; ‡‡ICM - Índice de Choque Modificado; †AUC - area under the curve;

** IC - intervalo de confiança; ††Estatisticamente significativo.

* AUC - Area Under the Curve;

** ROC - Receiver Operating Characteristic.

**Figura 1:** Comparação das áreas sob a curva (AUC) das curvas ROC do Índice de Choque e do Índice de Choque Modificado para predição de mortalidade intra-hospitalar em pacientes com diagnóstico de choque, Porto Alegre, Rio Grande do Sul, Brasil

## DISCUSSÃO

A comparação entre o Índice de Choque (IC) e o Índice de Choque Modificado (ICM) demonstrou que ambos possuem capacidade de predizer a mortalidade intra-hospitalar em pacientes com choque admitidos no Serviço de Emergência, porém o ICM apresentou desempenho mais consistente, especialmente nos tipos de choque mais prevalentes, respondendo ao objetivo central deste estudo.

A literatura ainda é incipiente quanto aos dados sobre a incidência de choque. Um estudo de coorte conduzido em um Serviço de Emergência na Tailândia observou que a incidência de choque foi de 876 de 113.651 indivíduos (7,7/1.000 visitas), sendo o choque séptico o mais prevalente, seguido do choque hipovolêmico e do choque cardiogênico. O desfecho da mortalidade intra-hospitalar foi observado em dois momentos, 7,9% dos óbitos ocorreram em até 7 dias, e 20,4% em 90 dias. Ainda, neste último momento, foram observados maiores índices em pacientes que tiveram choque hipovolêmico, seguido de choque cardiogênico e choque séptico^(10)^. A incidência e a taxa de mortalidade intra-hospitalar observadas neste estudo foram superiores às descritas na literatura internacional, o que pode ser parcialmente explicado pelo perfil da instituição onde a pesquisa foi conduzida. Trata-se de um hospital universitário que atende majoritariamente pacientes do Sistema Único de Saúde (SUS), muitos dos quais apresentam barreiras de acesso aos serviços de saúde, com dificuldade de acompanhamento ambulatorial e menor oferta de cuidados preventivos e de monitorização contínua. Esse cenário contribui para que os pacientes cheguem à emergência em estágios mais avançados de deterioração hemodinâmica, frequentemente com múltiplas comorbidades descompensadas, infecções graves ou desidratação severa, aumentando a complexidade do atendimento e o risco de mortalidade.

Apesar das diferenças nos desfechos de mortalidade, a distribuição das etiologias de choque observada em nossa amostra é consistente com estudos prévios, reforçando que o choque séptico permanece como a principal causa em serviços de emergência, seguido por causas hipovolêmicas e cardiogênicas.

Adicionalmente, verificou-se que idosos, homens e indivíduos brancos apresentaram maior suscetibilidade ao desenvolvimento de choque. Dois estudos de coorte realizados em serviços de emergência, uma coorte retrospectiva realizada com 876 pacientes clínicos na Tailândia e outra coorte de 12 anos realizada na Dinamarca com 1.553 pacientes, constataram também que este perfil de pacientes é mais vulnerável a desenvolver choque^(10,11)^.

A predominância de indivíduos idosos nos serviços de emergência pode ser parcialmente atribuída ao processo de transição demográfica em curso no Brasil, caracterizado pelo aumento expressivo da população idosa. Esse grupo etário apresenta maior carga de comorbidades, maior predisposição a eventos cardiovasculares e maior vulnerabilidade a processos infecciosos, fatores que elevam substancialmente o risco de desenvolvimento de choque, bem como as taxas de mortalidade intra-hospitalar, o tempo de permanência e os custos relacionados à utilização de recursos assistenciais, diretos e indiretos^(12,13)^. Nesse contexto, o manejo inicial assume papel central, especialmente quando as manifestações clínicas se apresentam como queixas inespecíficas, exigindo do profissional a capacidade de reconhecer sinais precoces de instabilidade hemodinâmica e aplicar protocolos de avaliação e intervenção específicos^(14,15)^. Nos serviços que adotam sistemas de classificação de risco, destaca-se a importância do enfermeiro na identificação precoce dos fatores de risco, possibilitando a atribuição adequada do discriminador clínico, a priorização do atendimento e a implementação oportuna de intervenções que minimizem a progressão do choque e suas complicações.

O presente estudo demonstrou que o IC apresentou melhor capacidade preditiva para mortalidade intra-hospitalar nos choques misto e séptico, enquanto o ICM mostrou desempenho superior nos choques misto, séptico e cardiogênico. Em consonância com esses achados, uma coorte realizada com 200 pacientes em uma UTI italiana identificou maior mortalidade entre indivíduos com choque misto (53,1%) em comparação àqueles com choque de causa única (27,8%), atribuída à maior complexidade fisiopatológica^(16)^. No presente estudo, essa letalidade foi ainda mais elevada, atingindo 70% dos pacientes com choque misto. Para o choque cardiogênico, pesquisas anteriores descrevem mortalidade variando entre 27% e 51%^(17,18)^, valores inferiores aos encontrados em nossa amostra, possivelmente em razão de maior carga de comorbidades, fragilidade e menor reserva fisiológica, fatores que comprometem os mecanismos compensatórios necessários à reversão do choque refratário.

Apesar da relevância clínica desses achados, a literatura ainda carece de estudos que avaliem especificamente o desempenho dos escores IC e ICM no choque misto. Em pacientes com doenças cardiovasculares, evidências demonstram associação entre IC ≥ 0,8 e complicações graves, como o choque cardiogênico^(18)^, enquanto o ICM ≥ 0,9 tem se mostrado eficaz na predição de mortalidade em infarto agudo do miocárdio^(19)^. Contudo, os pontos de corte identificados neste estudo para melhor desempenho preditivo no choque cardiogênico foram superiores (IC ≥ 1,0; ICM ≥ 1,1), o que pode refletir diferenças nas características das populações analisadas, no perfil assistencial e na gravidade dos casos atendidos.

Da mesma forma, o choque séptico apresentou elevada mortalidade em nossa amostra. Estudos nacionais relatam letalidade de 53,5%^(20)^, enquanto no presente estudo o índice atingiu 80%. Pesquisas anteriores reportam eficácia dos escores IC e ICM com valores de corte de 0,76 e 1,35, respectivamente^(21)^, ou IC ≥ 1,06 e ICM ≥ 1,64^(22)^. Em contraste, os achados deste estudo indicaram ponto de corte mais baixo (≥ 1,0) como mais adequado para ambos os escores, possivelmente devido ao perfil clínico mais grave dos pacientes atendidos.

No choque hipovolêmico, os melhores pontos de corte observados foram ≥ 1,4 para o IC e ≥ 1,9 para o ICM. Embora estudos com populações vítimas de trauma sugiram IC > 0,9 como indicador de maior mortalidade^(5,23,24)^, análises com coortes mais amplas demonstram que IC > 1,4 está associado a desfechos desfavoráveis^(25)^. Em relação ao ICM, valores > 1,3 foram correlacionados a maior mortalidade em trauma^(26)^. Assim, as discrepâncias observadas entre os diferentes estudos podem ser explicadas por diferenças populacionais, etiológicas e contextuais.

A compreensão da fisiologia subjacente aos escores auxilia na interpretação de seu desempenho. O ICM incorpora a pressão arterial média, refletindo mais adequadamente o estado de perfusão tecidual em comparação ao IC. A queda precoce da pressão arterial diastólica, captada pelo ICM, antecipa a detecção de instabilidade hemodinâmica, conferindo maior sensibilidade para identificar estados compensatórios iniciais do choque^(27)^. Isso pode explicar o melhor desempenho global do ICM observado neste estudo, com exceção do choque hipovolêmico.

Por fim, esses achados destacam o potencial do ICM como ferramenta clínica relevante na avaliação de enfermagem. Sua incorporação à avaliação inicial ou aos protocolos de classificação de risco pode auxiliar o enfermeiro na identificação precoce de pacientes com risco aumentado de deterioração clínica, otimizando a priorização do atendimento e a tomada de decisão desde a classificação de risco. Além disso, este estudo se destaca por ser um dos primeiros a avaliar comparativamente a performance do IC e do ICM em uma coorte ampla de pacientes brasileiros atendidos em um serviço de emergência, reforçando a utilidade de ferramentas simples e de baixo custo em contextos de alta demanda e recursos limitados.

### Limitações do estudo

Este estudo apresenta limitações inerentes ao seu delineamento retrospectivo, especialmente a dependência da qualidade dos registros nos prontuários eletrônicos, o que pode ter introduzido viés de informação devido à ausência de dados em alguns casos. No entanto, o tamanho da amostra e a análise estatística robusta conferem relevância e consistência aos resultados obtidos. Recomenda-se cautela na generalização dos achados, sendo necessários estudos multicêntricos prospectivos para validar os pontos de corte identificados e confirmar a aplicabilidade desses achados em diferentes contextos clínicos.

### Contribuições do estudo

Os resultados desta pesquisa contribuem para o fortalecimento da tríade ensino, assistência e pesquisa, ao promover a formação de estudantes e profissionais, ampliando o entendimento sobre o uso de escores preditivos no contexto da prática de enfermagem. Além disso, oferecem implicações práticas para a enfermagem em serviços de emergência ao demonstrarem que esses escores podem ser incorporados como ferramentas rápidas para triagem e estratificação de risco, facilitando o raciocínio clínico, a priorização de cuidados e a alocação eficiente de recursos. Esses achados também podem impulsionar o desenvolvimento de fluxogramas, protocolos e políticas públicas voltados à redução da mortalidade por choque, além de servir como base para futuros estudos de validação em contextos clínicos e populacionais diversos.

## CONCLUSÕES

O Índice de Choque Modificado (ICM) demonstrou superioridade na predição da mortalidade intra-hospitalar em pacientes admitidos com choque, especialmente nos casos de choque séptico, cardiogênico e misto. Em contrapartida, o Índice de Choque (IC) apresentou melhor capacidade preditiva apenas no choque hipovolêmico. Esses resultados destacam o potencial dos escores, em particular do ICM, como ferramentas simples e eficazes para triagem e estratificação de risco em serviços de emergência. O uso desses escores pode apoiar o raciocínio clínico na enfermagem, facilitar a tomada de decisões rápidas e contribuir para a priorização do cuidado em contextos de alta demanda e recursos limitados. Recomenda-se a incorporação desses escores em protocolos assistenciais, bem como a realização de novos estudos multicêntricos e prospectivos para validar os pontos de corte identificados e expandir a aplicabilidade dos índices de choque em diferentes cenários clínicos.

## Data Availability

Os dados de pesquisa só estão disponíveis mediante solicitação.
